# Early substance consumption and problematic use of video games in adolescence

**DOI:** 10.3389/fpsyg.2015.00501

**Published:** 2015-04-28

**Authors:** Adélaïde Coëffec, Lucia Romo, Nathalie Cheze, Hélène Riazuelo, Sophie Plantey, Gayatri Kotbagi, Laurence Kern

**Affiliations:** ^1^CLIPSYD, EA4430, UFR de Sciences Psychologiques et Sciences de l’Education, Université de Paris Ouest Nanterre La Défense, Nanterre, France; ^2^Service d’addictologie de l’hôpital René Muret, Sevran, France; ^3^La Clinique des Maladies Mentales et de l’Encéphale, Centre Hospitalier Sainte-Anne, Paris, France; ^4^Centre de Psychiatrie et Neurosciences, INSERM U894, Paris, France; ^5^Laboratoire Modal’X, Université de Paris Ouest Nanterre La Défense, Nanterre, France; ^6^CeRSM, EA 2931, UFR STAPS, Université de Paris Ouest Nanterre La Défense, Nanterre, France; ^7^Centre Hospitalier Jean-Martin Charcot, Plaisir, France

**Keywords:** problematic usage, adolescence, video gaming, alcohol, tobacco, cannabis

## Abstract

Substance use as well as use of video games is frequent among young people. The purpose of this research was to study the links between the use of video games and the consumption of various substances such as alcohol, tobacco or cannabis at adolescence. In order to do so, 1423 students from middle and high schools filled an auto-questionnaire that included questions on age, gender, year of study, use of video games and consumptions of alcohol (Alcohol Use Disorders Identification Test Short version, AUDIT-C), tobacco (Heaviness of Smoking Index, HSI), and cannabis (Cannabis Abuse Screening Test, CAST). We found that 92.1% of teens use video games and 17.7% have a problematic use of video games (PUVG). Furthermore, results show that substance consumption seems frequent with 19.8 and 8.3% of participants having hazardous alcohol and cannabis consumptions respectively and 5.2% having a moderate to high tobacco dependence. Video gamers consumed significantly more alcohol and gamers with PUVG started their substance consumption (alcohol, tobacco, and cannabis) earlier. PUVG was found to be negatively correlated to age at first substance consumption, but positively correlated to the time spent playing video games. However, it was not correlated to risks of substance dependence (scores of AUDIT-C, HSI, and CAST). Finally, our results are consistent with the literature, in regard to frequency of substance use and use of video games in adolescence. These data will allow for a better consideration of prevention strategies and future care in this particular field.

## Introduction

Experimenting with psychoactive substances, such as alcohol, tobacco or cannabis, is common during adolescence (Currie et al., 2010; Spilka et al., 2012a,b; Spilka and Le Nézet, 2013). Playing video games is also popular in this population and in some cases, can become problematic, especially if coexistent with other psychopathological dimensions. Several other studies have found associations between video games and substance consumption. While some studies focus on the links between gambling, chance games and substance consumption (Lane et al., 2004; Phillips and Ogeil, 2007), others highlight the associations between video games and substance consumptions (Wenzel et al., 2009; Armstrong et al., 2010; Denniston et al., 2011; Raiff et al., 2012; Walther et al., 2012; Van Rooij et al., 2014). However, until this day and to our knowledge, very few French studies have investigated the possible links between substance consumption and use of video games. Thus, the purpose of this study was to explore the links between the use of video games and consumption of alcohol, tobacco and cannabis in a population of adolescents. The underlying premise of our study is that, there exists a profile (personality and psychopathological) common to players with problematic use of video games (PUVG; i.e., according some researchers players addicted to video games) and young video gamers who are at risk of developing a PUVG.

## Literature Review

### Substance Consumption Amongst Adolescents

An HBSC survey (Health Behaviour in School-aged Children, 2010) carried out on a young population aged between 11 and 16 years old, shows that alcohol, tobacco and cannabis intake are quite frequent and increases with age. Thus, among the students of the sixth grade, 59.3, 12.7, and 1.5% had already consumed alcohol, tobacco and cannabis, respectively. Moreover, 6.8% of sixth graders and 34% of ninth graders have reported getting drunk (Currie et al., 2010; Spilka et al., 2012a).

According to the ESCAPAD survey (2011) (or in French: “Enquête sur la Santé et les Consommations lors de l’Appel de Préparation A la Défense”), approximately 75% of 17-year-old youngsters declare having consumed alcohol, 42% declare having consumed tobacco (Spilka et al., 2012b) and 26.8% declare having consumed cannabis (Spilka and Le Nézet, 2013) during the past month.

### Use of Video Games

Use of video games is common amongst children and adolescents (Tejeiro and Moran, 2002; Wood et al., 2004). In fact, Le Heuzey and Mouren (2012) found that 87% of 11–13 year-olds and 80% of 15–17 year-olds play a video game at least once a day. The most investigated video games by researchers are online gaming platforms, specifically the MMORPG (massively multiplayer online role-playing game). These games have features that make them very attractive for teens, such as: anonymousness, accessibility, user-friendliness, generates excitement, interactivity, competitiveness, immersion, abundance of information, and in certain cases, a persistent virtual universe (Greenfield and Ceap, 1999; Griffiths and Wood, 2000; Morahan-Martin and Schumacher, 2000; Chou et al., 2005; Ng and Wiemer-Hastings, 2005; Allison et al., 2006; Sebeyran, 2008; Minotte, 2010; INSERM, 2014).

The notion of “addiction to video games” is not yet an established disorder. Nevertheless, “Internet Gaming Disorder” has been introduced in the section III of the DSM-5 (Diagnostic and Statistical Manual of Mental Disorders, version 5, American Psychiatric Association, 2013). Most studies that analyze online usage of video games use the broader term of “cyberdependence” (Varescon, 2009). However, the notion of “problematic use” is beginning to spread in the scientific field, as evidenced by INSERM’s (2014) report, which includes a definition that is very similar to Goodman’s (1990) criteria of addiction. Despite the fact that PUVG is poorly defined, often covering notions of abusive, excessive or addictive use of video games, it was found to have a prevalence ranging between 1.3% to over 50%. This wide variability between studies could be explained by the use of different assessment instruments, selection bias, inclusion criteria such as age, etc. (Haagsma et al., 2012; Festl et al., 2013; King et al., 2013; Pápay et al., 2013; Ricquebourg et al., 2013).

### Video Games and Substance Use

The majority of studies find a link between substance use and video gaming habits. A recent research shows that boys consuming tobacco, cannabis and alcohol double their risk of having a high level of PUVG (assessed by PVG or “problematic video gaming”) than the ones who did not (Van Rooij et al., 2014).

When it comes to alcohol consumption, it seems to be the most associated to the time spent playing video games. In fact, Armstrong et al. (2010) showed, in a sample of 4691 elementary school students, that the time spent in front of a television screen and playing video games is positively correlated to alcohol consumption.

When it comes to age at first substance consumption, initiation to alcohol use before the age of 13 was found to be significantly correlated to frequent use of television, computers and video games (Denniston et al., 2011).

When it comes to substance abuse and dependence, the prevalence of alcohol abuse was found to increase with time spent playing video games in a Norwegian population varying from 16 to 74 years old (Wenzel et al., 2009). In addition, individuals that report being the most addicted to substances such as alcohol, caffeine, chocolate and cigarettes, are the ones who are the most vulnerable to be dependent on another activity such as exercising, watching television, playing chance games, gambling, using internet and video games (Greenberg et al., 1999). Thus, PUVG was associated with the risks of alcohol, tobacco and cannabis dependencies (Ream et al., 2011a,b).

Interestingly, Raiff et al. (2012) showed that, in an adult population, the use of video games is more frequent and longer in smokers than non-smokers.

However, some studies failed to find a relation between the time spent playing video games and consumption of certain substances. Thus, Walther et al. (2012) showed that except for the use of cannabis, neither tobacco nor alcohol consumption, co-occurs with video games. In addition, McClure and Mears (1986) showed that the students (*n* = 190) who played video games daily did not consume more alcohol or tobacco than those who played once or twice per month.

## Methodology

### Population

Our sample consisted of 1423 French students, aged between 11 and 17 years, recruited from middle and high schools (7th, 8th, 10th, and 11th grade) in the region of “Ile de France.” The students were informed about the objectives of our study and participated voluntarily by completing an auto-questionnaire during school hours. The parents were also informed about their rights to refuse their children’s participation in this study. This study followed the recommendations given by the committee of ethics of the Psychological Science and Learning Science department, University of Paris Ouest Nanterre La Défense, UFR SPE (Department of Psychology and Education). The education authority of Versailles, the participating schools and the higher education establishments also accepted the questionnaire.

### Measures

The auto-questionnaire consisted of two parts: the first included general questions about gender, age and level of study. The second was divided into four sections:

#### Use of Video Games

The PUVG questionnaire was used. In order to assess PUVG, this questionnaire was established in reference to the criteria for substance dependence in the DSM-IV-TR (Diagnostic and Statistical Manual of Mental Disorders, version IV-TR, American Psychiatric Association, 2000). This measure for PUVG was used, since no valid instrument in French existed at the beginning of our study. The total score ranges from 0 to 7 points. A score equal or superior to three indicates a PUVG. This cut-off score is the same as that used to diagnose substance dependency. The subject was asked to specify the time spent playing video games on days with and without school. Participants were classified as “video gamers” if they reported playing video games in the last 12 months. It should be noted that gaming devices and the types of video games (arcade, console or computer-based games, online gaming, etc.) were also explored in this study.

#### Alcohol Consumption

The AUDIT-C (Alcohol Use Disorders Identification Test Short version; Bush et al., 1998) was used. This instrument allows a pertinent evaluation of hazardous alcohol consumption, over the past 12 months, through three items on frequency, quantity and frequency of drunkenness (six or more drinks on a single occasion). It was used in various studies to identify hazardous alcohol consumption (Bush et al., 1998; Gual et al., 2002), including epidemiological studies such as the Health barometer (in French: “Baromètre de Santé”), but does not have a French validation known to date. A score equal or superior to four for males and equal or superior to three for females indicates alcohol consumption at risk of becoming dependent (Bradley et al., 2007).

#### Tobacco Consumption

The HSI (Heaviness of Smoking Index), which allows a quick detection of current tobacco dependence (abridged version of *Fagerström Test for Nicotine Dependence* by Fageström, 1978) was used. Despite the fact this questionnaire was only validated for adults (Heatherton et al., 1989), it has been previously used in adolescent populations (Hastier et al., 2006). Despite its weak psychometric qualities (internal coherence between 0.62 and 0.65), it is still widely used for its rapidity in administration (Etter, 2005). A total score, given by the two items of this questionnaire, between: 0 and 1 shows little or no dependence; 2 and 3 shows moderate dependence; 4 and 6 shows strong dependence (Fagerström et al., 1990).

#### Cannabis Consumption

The CAST (Cannabis Abuse Screening Test) was used. This test was validated in France by a survey called *ADOTECNO* (ADOlescents et TEChniques d’évaluation des consommations Nocives) on 1728 students. It was also validated in the general population by Legleye et al. (2007). A score equal or superior to two indicates a risk of cannabis dependence.

### Statistical Analysis

SPSS 19 was used for all statistical analyses. Descriptive analyses (such as percentages, means and standard deviations) were carried out in order to describe the sample population. Subsequently, we carried out bivariate analyses (Student’s *t*-test, Chi-square, Pearson’s correlation) in order to investigate the possible links between the variables. Finally, multivariate analyses (MANOVA, regression) were carried out in order to highlight the variables, which had a significant statistical weight on PUVG while taking them all into account. Thus, we were able to highlight the percentage of variance explained and trace profiles of “video gamers” with PUVG.

## Results

### Video Gaming

A total of 92.1% (*N* = 1289) were considered video gamers against 7.9% (*N* = 111) non-video gamers. Among the players who replied to the PUVG questionnaire (*N* = 1192), 17.7% (*N* = 211) presented a PUVG (with three or more points). Only 1.1% of them exhibited all the criteria (maximum score of 7 points). Time spent playing video games on a school day was significantly lower than on a day without school [53 min (SD: 1 h 23 min) and 2 h 12 min (SD: 2 h 45 min) respectively; *t*(1288) = 22.78, *p* < 0.0001]. Moreover, video gamers with PUVG spend more time on video games than video gamers without PUVG [on days with school (*t* = 10.62, *p* < 0.0001)] and without school [*t*(1085) = 10.8, *p* < 0.0001].

When it comes to gaming devices and types of video games: the three that were most cited in the category of gaming devices are, computers (85.1%), fixed consoles (75.2%), and cell phones (66%). As for the types of games, they are, racing games (54.8%), platform games (48.9%) and sports games (48.3%).

### Consumptions

Around half of our population (46.7%) declared consuming alcohol. The mean age at first consumption was 12.4 years (SD = 2.7). We noticed that 89.3% of first consumptions took place after the age of 8. In other words, one out of 10 adolescents had tasted alcohol for the first time before the age of 8. Moreover, 19% have an at-risk consumption of alcohol.

Approximately 16.7% of our population declared smoking cigarettes. The average starting age reported was 12.8 years (SD = 1.9). We found that 90.3% of smokers started smoking at the age of 10 or above. Moreover, 5.2% have a high tobacco dependency.

A total of 21.1% of students declared having smoked cannabis at least once in their lifetime and 8.3% have had hazardous cannabis consumption, while 8.6% declared currently smoking.

### Comparison between Video Gamers and Non-Video Gamers

Video gamers have a significantly higher consumption of alcohol than non-video gamers (*p* = 0.04). No other significant differences were found between the two groups for the variables presented in Table 1. Table 2 describes the results on the scales measuring risk of dependence and first age of consumption for video gamers and non video gamers.

**Table 1:** **Percentage (%) of consumers and subjects at risk of dependence**.

	% of subjects who have consumed alcohol	% of subjects who are at risk of dependence	% Smokers	% of subjects whose total score indicates			% of subjects having smoked cannabis at least once	% of subjects currently smoking cannabis	% of subject at risk of cannabis dependence
				No	Moderate dependence	Strong			
Non-video gamers (*N* = 111)	36.8	16	21.1	96.3	3.7	0	26.5	8.9	9.9
Video gamers (*N* = 1289)	45.1	20.3	16.4	94.5	3.9	1.6	20.7	9.1	8

**Table 2:** **Results of AUDIT-C, HSI, and CAST, and mean age at first consumption**.

	Mean (SD)					
	Total Score of			Age at first consumption of		
	AUDIT-C	HSI	CAST	Alcohol	Tobacco	Cannabis
Non-video gamers (*N* = 111)	1.06 (2.28)	0.14 (0.52)	0.40 (1.11)	12.7 (2.7)	13.4 (1.4)	14.3 (1.8)
Video gamers (*N* = 1289)	1.49 (2.31)	0.19 (0.75)	0.29 (0.90)	12.3 (2.7)	12.7 (1.9)	14.3 (1.5)

Factorial ANOVA was performed in order to study the time spent playing video games on a day with as well as without school for those who consume alcohol, cannabis and tobacco versus those who do not. The ANOVA was significant. Bonferroni’s *post hoc* tests showed significant difference uniquely for the group of smokers and non-smokers with respect to time spent playing video games on a day of school (*n* = 1199, *p* = 0.004) as well as on a day without school (*n* = 1199, *p* = 0.003). Smokers were found to spend more time playing video games.

We also conducted a MANOVA with the dependent variables AUDIT-C, HSI, and CAST comparing the two groups of video gamers and non-video gamers. The MANOVA shows overall substantial differences between the two groups. [*F*(3,1196) = 3.3, *p* = 0.02]. In particular, video gamers have a higher score on AUDIT-C (Bonferroni *post hoc* tests, *p* = 0.106) and on CAST (Bonferroni *post hoc* tests, *p* = 0.207) than non-video gamers. However, no difference was found when analyzing the score on HSI (*p* = 0.538).

We also conducted a MANOVA with the dependent variables age at first consumption of alcohol, tobacco and cannabis comparing the two groups of video gamers and non-video gamers. However, no significant differences were found between these two groups [*F*(3,153) = 0.965, *p* = 0.41] for these variables.

### Correlational Analysis

Table 3 displays the correlations between the PUVG score; mean scores of AUDIT-C, HSI, and CAST and age at first substance consumption.

**Table 3:** **Pearson’s correlations between PUVG score, time spent playing video games, score of AUDIT-C, HSI, CAST and age at first substance consumption**.

	Score of PUVG (*N*) *r* (*p* if significatif)	Time spent playing video games on a school day (*N*) *r* (*p* if significatif)	Time spent playing video games on a day without school (*N*) *r* (*p* if significant)
Score of AUDIT-C	(1075) 0.04	(1152) 0.11 (*p* = 0.0002)	(1153) 0.07 (*p* = 0.01)
Age at first consumption of alcohol	(531) –0.12 (*p* = 0.005)	(574) –0.07	(574) –0.10 (*p* = 0.02)
Score of HSI	(1171) 0.05	(1266) 0.08 (*p* = 0.004)	(1266) 0.10 (*p* < 0.001)
Age at first consumption of tobacco	(268) –0.19 (*p* = 0.001)	(289) –0.19 (*p* = 0.001)	(288) –0.2 (*p* < 0.001)
Score of CAST	(1134) 0.04	(1222) 0.08 (*p* = 0.008)	(1222) 0.07 (*p* = 0.012)
Age at first consumption of cannabis	(234) –0.28 (*p* < 0.001)	(245) –0.31 (*p* < 0.001)	(245) –0.30 (*p* < 0.0001)
Score of PUVG	–	(1085) 0.38 (*p* < 0.001)	(1085) 0.4 (*p* < 0.001)
Time spent playing video games on a school day	–	–	(1288) 0.68 (*p* < 0.001)

If we take into account only those who smoke (*N* = 100), we notice a significant correlation between the score on PUVG and HSI (*r* = 0.19, *p* = 0.052). Similarly, if we consider those who consume cannabis (*N* = 150) we observe significant and positive correlation between their score on PUVG and CAST (*r* = 0.27, *p* = 0.001).

### Multiple Linear Regressions

We tested a linear regression model in an attempt to explain the score of PUVG (dependent variable) by the scores of AUDIT-C, HSI, CAST, age at first substance(s) consumption(s) and time spent playing video games (on a day with/without school; independent variables). It should be noted that this analysis included a smaller sample than the initial one, consisting of 108 subjects who consume or have consumed the three substances. This regression model explained 37.76% (*R*^2^ = 0.3776) of the variability of PUVG score. The significant descriptive variable in this model are time spent playing video games on a day with school (b* = 0.311; *p* = 0.018), age at first consumption of cannabis (b* = –0.25; *p* = 0.02) and age at first consumption of tobacco (b* = 0.22; *p* = 0.04) (Table 4).

**Table 4:** **Multiple regressions with PUVG score as dependent variable**.

*N* = 108	b*	Standard error b*	*t*	*p*
(Constant)			1.68	0.09
Score of AUDIT-C	–0.08	0.09	–0.86	0.39
Score of HSI	0.17	0.09	1.82	0.07
Score of CAST	0.17	0.09	1.83	0.07
Age at first consumption of alcohol	–0.09	0.09	–1.02	0.31
Age at first consumption of tobacco	0.22*	0.11*	2.06*	0.04*
Age at first consumption of cannabis	–0.25*	0.10*	–2.34*	0.02*
Time spent playing video games on a school day	0.31*	0.13*	2.41*	0.017*
Time spent playing video games on a day without school	0.2	0.13	1.59	0.11

We also carried out a step-by-step ascending regression (*R*^2^ = 0.367). Time spent playing video games on a school day (b* = 0.292) and age at first consumption of cannabis (b* = –0.26; *p* < 0.05) as well as, the score on HSI (b* = 0.148), time spent playing video games on a day without school (b* = 0.191), score on CAST (b* = 0.153) and age at first consumption of tobacco (b* = 0.2; *p* > 0.05) were the variables, which were selected by this method.

Similarly, we also carried out a step-by-step descendant regression (*R*^2^ = 0.32). Time spent playing video games on a school day (b* = 0.446), age at first consumption of cannabis (b* = –0.18) and score of HSI (b* = 0.173; *p* < 0.05) were the variables, which were selected by this method.

Thus, time spent playing video games on a school day and an earlier age of first consumption of cannabis increase the risks of having a PUVG. These two variables were found to be significant in all the regression models that were tested (regression, ascendant, and descendant).

## Discussion

### Prevalence

In our adolescent population (*N* = 1423), we found 92.1% of video gamers (*N* = 1289) and 17.7% of video gamers with PUVG (*N* = 211). Substance use (alcohol, tobacco, and cannabis) in this population was frequent, which goes in accordance with the epidemiological data in the literature (Currie et al., 2010; Spilka et al., 2012a). Therefore, 8.3 and 19.8% of teens present hazardous cannabis and alcohol consumptions respectively and 5.2% show a moderate to strong dependence to tobacco smoking. These percentages do not vary between video gamers and non-video gamers.

### Video Games and Substance Consumption

Video gamers have a significantly higher consumption of alcohol than non-video gamers. This was the only significant difference found between these two groups when it came to substance consumption. Our results are similar to those of McClure and Mears (1986) for tobacco consumption, but inconsistent with their results concerning alcohol consumption. It seems that exposure to tobacco and cannabis through video games is not as important as exposure to alcohol. In fact, video gamers had similar chances to have consumed one of these three substances than non-video gamers. Our results: (1) go against those of Barrientos-Gutiérrez et al. (2012) who show earlier initiation to tobacco smoking among youth exposed to media; (2) are consistent to those of Tucker et al. (2013) concerning an increased likelihood of alcohol use in young video gamers.

Moreover, our results (MANOVA) show that there exist at least two significant differences between video gamers and non-video gamers with respect to their score on substance dependence (alcohol, tobacco, cannabis). However, no significant differences were found with respect to age at first consumption for the two groups.

### Time Spent Playing Video Games and Substance Consumption

There were no significant differences in time spent playing video games between consumers of alcohol and cannabis and non-consumers. This time was significantly longer for tobacco smokers than non-smokers. These results are consistent with those of Raiff et al. (2012). Time spent playing video games (on a day with or without school) is positively correlated to the scores of AUDIT-C, HSI, and CAST. In addition, our results concerning alcohol consumption are: (1) in line with those of Wenzel et al. (2009) who found that alcohol abuse increases with time spent playing video games; (2) inconsistent with those of Armstrong et al. (2010), who found that drinking alcohol is more frequent among young people who spend more time playing video games.

It must be noted that longer time spent playing video games during a day without school is associated with an earlier age of first substance consumption (alcohol, tobacco, and cannabis). These results are in accordance with those of Denniston et al. (2011) who found that, an early initiation to alcohol (before 13 years old), is associated with a more frequent use of television, computers and video games. In addition, there was positive correlation between time spent playing video games on a school day and age at first consumption of cannabis and tobacco. However, this correlation could not be extended to the age at first alcohol consumption.

### PUVG and Substance Consumption

Previous studies have focused on finding a link or a co-occurrence between PUVG and substance use, as mentioned earlier. Our results are inconsistent with the literature (Greenberg et al., 1999; Ream et al., 2011a,b; Walther et al., 2012) since PUVG score was not found to be correlated to scores of AUDIT-C, HSI, or CAST.

Problematic use of video games seems to be associated with an earlier initiation of substances since there exists a significant negative correlation between PUVG score and age at first substance consumption (alcohol, tobacco, and cannabis). Hence, an earlier starting age is linked to an increased risk of PUVG. To our knowledge, these data have not been explored by other studies.

Furthermore, only 36.76% of the variability of PUVG score was explained by our regression model that include scores of AUDIT-C, HSI, CAST, age at first substance consumption and time spent playing video games (on a day with/without school) as independent variables. Age at first consumption of tobacco and of cannabis, time spent playing video games on a day with school explain the variance significantly. The two variables that stood out (despite the method of regression used) were, time spent playing video games on a school day and an earlier age of first consumption of cannabis.

### Limitations

It is to be noted that our study has some limitations; starting from the questionnaire measuring PUVG which is based on the DSM-IV-TR. Moreover, our sample was a convenience sample: recruitments were only carried out in schools that voluntarily accepted to participate in the study. Groups of video gamers versus non-video gamers and those with or without PUVG are not matched, which does not allow us to exclude or control some confounding variables, such as academic results. In fact, according to some authors (Chiu et al., 2004; Skoric et al., 2009), there exists a negative correlation between PUVG and academic performance. We could also mention weak family structures, which could lead to the risks of dependency (INSERM, 2014). We could also mention gender, as boys tend to play more than girls (INSERM, 2014).

In addition, a qualitative study based on hetero assessment would allow us to refine our results, which as of now are only based on auto-evaluations.

## Conclusion

Problematic use of video games and consumption of psychoactive substances (specifically alcohol use) are frequent at adolescence. Adolescents who play video games consume significantly more alcohol than non-video gamers. This was not the case for tobacco or cannabis consumption.

Despite the limitations of the instrument we used to assess PUVG, our results provide important information on the associations, poorly investigated, between playing video games and use of alcohol, tobacco, and cannabis in an adolescent population. Thus, age of first substance consumption (alcohol, tobacco, and cannabis) does not differ significantly between video gamers and non-video gamers but is negatively correlated to PUVG score and time spent playing video games on a day without school. The risk of developing a substance dependence (total scores of the AUDIT-C, HSI, and CAST) was not found to be correlated to PUVG score but was positively correlated to time spent playing video games. On the other hand, the age at first consumption of tobacco and of cannabis and the time spent playing video games on a day of school were significant predictors of PUVG.

An earlier age of substance consumption is associated with PUVG. Thus, it seemed important to point out that, although PUVG is potentially transient, substance consumption initiated earlier predisposes to the development of future dependences. Therefore, there is a need to highlight prevention and information on the appropriate use of new technologies by adolescents and young adults, and particularly the use of video games. However, our results only highlight the possible links between the variables and do not indicate the direction of the relationship between the variables. Longitudinal studies with more robust tools are necessary in order to throw light upon the possible underlying mechanisms.

### Conflict of Interest Statement

Our team of research has received funding from the gambling industry operators (FDJ and PMU) and IREB (Institut de Recherches Scientifiques sur les Boissons—“Institute for Scientific Research on Drinks”).
